# Randomized Controlled Trial of the Meaning‐Making Intervention (MMi) in Patients Newly Diagnosed With Advanced Cancer: Full Trial

**DOI:** 10.1002/pon.70421

**Published:** 2026-03-19

**Authors:** Melissa Henry, S. Robin Cohen, Daren K. Heyland, Robert Platt, Xun Zhang, Walter Gotlieb, Susie Lau, Carol‐Ann Vasilevsky, Michael Hier, Nader Sadeghi, Khalil Sultanem, Gerald Batist, Margarida Costa, Clara Bolster‐Foucault, Cassy Shitong Wang, Jason Agulnik

**Affiliations:** ^1^ McGill University Montreal Quebec Canada; ^2^ Jewish General Hospital Montreal Quebec Canada; ^3^ Lady Davis Institute for Medical Research Montreal Quebec Canada; ^4^ Queen's University Kingston Ontario Canada; ^5^ McGill University Health Centre Montreal Quebec Canada

**Keywords:** advanced cancer, anxiety, depression, existential, intervention, meaning in life, meaning‐making, oncology, post‐traumatic growth, psychological

## Abstract

**Background:**

This study aimed to test whether the Meaning‐Making intervention (MMi) increases the sense of meaning in life in people newly diagnosed with any type of advanced cancer.

**Methods:**

We conducted a 3‐arm parallel randomized controlled trial with 239 patients newly diagnosed (< 6 months) with advanced cancer (stages III or IV), assigned to either an experimental group (*n* = 80), an attention‐control group (*n* = 80), or a usual care control group (*n* = 79). Meaning in life (primary outcome), anxiety and depression, quality of life, existential wellbeing, and posttraumatic growth were measured at 2 months post randomization with follow‐up at 4 and 6 months.

**Results:**

There were no significant (*p* < 0.05) inter‐group differences in FACIT‐Sp‐12 Meaning subscale scores 2 months post‐randomization in independent two‐sample *t*‐tests (experimental group vs. usual care *p* = 0.65; experimental group vs. attention control *p* = 0.94), nor at 2, 4, and 6 months post‐randomization using a mixed effect linear regression model and adjusting for baseline characteristics and random effect of time (*p* = 0.55–0.99). In exploratory analyses, stage III experimental group participants seemed to present higher post‐traumatic growth on the PTGI 2 months post‐randomization than patients in the AC (*p* = 0.02), but this was not significant when applying a Bonferroni correction for multiple comparisons.

**Clinical implications:**

In this three‐arm randomized controlled trial, the MMi did not produce improvements on the primary or secondary outcomes compared with AC or UC. There may be some indications of a signal for benefit for patients with stage III cancer, which warrant follow‐up but cannot be considered definitive. Future work should prioritize targeting, dose, timing, and contextual moderators to clarify when, and for whom, meaning‐focused approaches are most effective.

## 1 Background

Meaning and meaninglessness are important existential concerns in advanced cancer and crucial in determining quality of life (QoL). These concerns intensify at time of diagnosis, disease progression, and with increased physical symptoms [1, 2]. While a loss of meaning can be part of a normal traumatic reaction to a life‐threatening event such as cancer, it can also characterize a more persistent state of demoralization, characterized by hopelessness, helplessness and existential despair/meaninglessness [3].

Psychosocial interventions are effective for people with cancer [1, 2]. However, most neglect existential issues such as meaning in life [4]. We define meaning as an existential construct with four different dimensions: meaning of events such as the cancer diagnosis (situational meaning; e.g.“What is the meaning of my cancer?”), meaning of my individual life (global meaning; e.g.“What is the meaning and purpose of my life now that I have cancer?”), meaning of life in the general, abstract sense (existential meaning; e.g.“What is the meaning of life and death?”) [5, 6, 7, 8, 9], and historical meaning: the unique way a person relates to the disease and treatments as a function of past challenges and experiences [10].

Meaning‐making interventions can buffer stress by facilitating the creation of new narratives, opening an alternative self and world perspective more congruent with the current situation [8]. Meaning can facilitate psychological adaptation to cancer, promote posttraumatic growth (i.e., finding benefits in the cancer diagnosis, which has been equated to positive meaning) and foster resilience, enhancing overall QoL and engagement in living their lives fully while facing a potentially life‐limiting disease [5, 6, 7, 8, 9]. This is especially important where a poor prognosis and physical deterioration challenge common ways of finding meaning and living fully, as well as considering people living longer with advanced cancer as treatments improve over time. This adaptation process can be influenced by pre‐diagnosis variables, including past life events and experiences, as well as sociocultural factors (e.g., social support, values, norms and beliefs) [9].

Several interventions were developed and empirically tested to address the existential plight of people with cancer. All provide a supportive environment for exploring existential dimensions such as authentic living, fear of death and dying, meaning of life, life priorities and goals, dignity, existential aloneness, and transcendence. These interventions differ in level of structure, didactic approach, number of sessions (6–24 weeks), philosophical grounding in existentialism, religious focus, and use of humanistic versus cognitive‐behavioral frameworks [1, 2]. Overall, trials suggest that meaning‐oriented therapies are effective but often face high dropout rates and/or lack ideal control conditions. Individual interventions flexible in timing and involving fewer sessions are generally the most feasible [1, 2].

The Meaning‐Making Intervention (MMi) is a brief, individualized, and manualized therapeutic approach based on post‐trauma literature, designed to facilitate a search for meaning following a cancer diagnosis [11]. Its one‐on‐one individualized approach can permit an in‐depth search for meaning and profound outcome, as patients have more time to individually work on the meaning of their cancer, of their life, and of life as a function of their past. While this historical meaning underlies patients' reactions to cancer and its treatment, patients are often unaware of it. With the exception of Breitbart et al. (2012) [12] and Lo et al. (2014) [13], other existential interventions tend to focus on the here‐and‐now. Furthermore, the MMi's brief (3‐4 sessions over one month compared to sessions over 7–24 weeks) and individualized format may facilitate attendance among patients with advanced cancer, through greater scheduling flexibility, accommodating physical decline through home‐based delivery, and providing a consistent therapeutic space tailored to the person. In palliative psychotherapy, group‐based interventions have been noted to pose challenges for some patients, especially when group composition changes over time or with repeated exposure to clinical deterioration in other participants, which can disrupt continuity and add emotion burden. In this care, time‐limited individual interventions may represent a more acceptable option for certain patients [14]. Through fostering a therapeutic alliance, the intervenor encourages self‐exploration, and addresses different levels of meaning (situational, global, existential, and historical) [9]. A small two‐arm randomized controlled trial (RCT) conducted with people newly diagnosed with cancer, mostly in early stages, found that the MMi improved self‐esteem, optimism, and self‐efficacy [11]. However, the study provided only immediate follow‐up (24 h post‐MMi) and did not evaluate effects on meaning, psychological distress, or on people with advanced cancer.

The current study addresses meaning using the MMi, a short‐term individualized approach, coupled with scientifically based tools and methods, including both usual care (UC) and attention‐control (AC) groups. This latter strengthens the design compared to other studies, which lack one of these control groups, considered the gold standard in RCTs of psychological interventions [15].

### 1.1 Research Questions and Hypotheses

This study aimed to evaluate whether adding the MMi to usual care enhanced meaning in life among people newly diagnosed with advanced cancer, compared to those receiving UC plus meetings with an empathic listener and UC alone. In this study, meaning in life refers to levels of perceived meaning and peace, as captured by the FACIT‐Sp‐12 Meaning/Peace subscale, reflecting greater existential coherence, purpose, and inner peace in the face of a life‐threatening diagnosis. The *primary research question* was: Does adding the MMi to usual care (experimental group or EG) enhance meaning in life among people newly diagnosed with advanced cancer, compared to those receiving [1]: usual care plus meetings with an empathic listener (AC group) and [2] UC alone at 2 months post‐randomization. *Primary hypothesis:* At 2 months post‐randomization the EG will report significantly more meaning in life [16, 17] than an AC group and UC group. The primary endpoint (2 months) was selected to ensure a sufficient retention rate (standard in RCT studies in advanced cancer) with the need to consider increases in effect with time as the MMi may prepare people to live life meaningfully in the face of disease progression. *Secondary hypotheses:* Compared to the AC and UC, the EG will report significantly higher existential wellbeing [18, 19, 20]; lower levels of anxiety and depression [21, 22]; higher QoL; more post‐traumatic growth [23]. *Secondary analyses:* We assessed sustained MMi effects on all outcomes at 4‐ and 6‐months post‐randomization, hypothesizing maintained effects. *Exploratory analyses:* To generate hypotheses for future research we planned to explore who benefited most from the MMi, through studying baseline effect modifiers such as cancer stage, sex, degree of perceived life‐threat, initial meaning in life, psychological distress, and physical wellbeing.

## 2 Methods

### 2.1 Trial Design

We conducted a full 3‐arm RCT (ClinicalTrial.gov Identifier NCT02583932). The EG received usual care plus MMi; the AC, usual care plus meetings with an empathic person; and the UC control, usual care alone. The AC arm aimed to understand whether any positive effects of the MMi were due to the intervention itself or reflect a by‐product of the broader basic therapeutic relationship, attention, or expectancy [15]. While common factors in psychotherapy (including the therapeutic relationship) are an important source of variability in any intervention's efficacy [24], the MMi is designed to specifically enhance people's meaning in life. Outcomes were evaluated using self‐administered questionnaires at baseline, 2, 4 and 6 months post‐randomization, with 2 months (approx.1 month post‐intervention) as primary endpoint since this was the most feasible timepoint in the pilot study (i.e., had the highest retention rates). Data from 60 participants in a pilot trial (EG = 19; AC = 21; UC = 20) were rolled into the current full trial since there was no change in design and outcomes were not analyzed (ClinicalTrials.gov #NCT01693991).

### 2.2 Trial Setting

Participants were recruited through research assistants (RAs) present in oncology clinics at two tertiary care hospitals in Montreal, Canada. RAs determined eligibility, kept a Record of Consecutive Patients, explained the study, and sought written free and informed consent. Eligible people received a study pamphlet, also available in oncology waiting rooms. Other recruitment strategies included posted ads in recruiting clinics.

### 2.3 Eligibility Criteria

*Participants were eligible if they were*: (1) diagnosed with advanced cancer stage III or IV—TNM classification system [25]—first occurrence, progression, or recurrence of any type of solid tumor < 2 months at referral and < 4 months at randomization; (2) physically able and willing to participate in weekly MMi or AC sessions; (3) > 18 years old; (4) alert and capable of giving free and informed consent according to referring clinician; and (5) able to speak and read English or French.

*Exclusion criteria included*: (1) Karnofsky Performance Status (KPS) [25, 26] score < 60 (prognosis indicator rated by referring oncologists/nurses or Research Coordinator) or expected survival < 6 months according to clinical judgment; (2) currently experiencing severe radiotherapy (Rx) side‐effects i.e., score of 3 or 4 on any site‐specific toxicity markers of the Eastern Cooperative Oncology Group (ECOG) Common Toxicity Criteria [27, 28] or with > 3 markers with score of 2 (moderate side‐effects), evaluated by the treating physician (in consultation with the radiotherapist); (3) currently suicidal score ≥ 2 on Beck Depression Inventory suicide item [29]. These people were referred to psychosocial services for a crisis‐oriented intervention; (4) known diagnosis of schizophrenia or schizoaffective disorder; (5) planning a trip within 2 months that would interrupt intervention delivery; (6) current or former therapy with MH.

### 2.4 Intervention and Comparator

#### 2.4.1 Meaning‐Making Intervention (MMi)

The MMi combines narrative storytelling with a “Lifeline” exercise, inviting people to broaden their perspective on the cancer diagnosis and integrate it into their whole life history, including past, present, and future. Sessions are structured around three main meaning‐making tasks. (1) The first step in the intervention is to look at how the cancer diagnosis affects the person's feelings and what it means to them. Patients are invited to explore their understanding of the cancer diagnosis and its impact on their lives and significant others, allowing work around illness‐related losses (e.g., health, employment, roles) and shattered beliefs (e.g., sense of health, control, self‐worthiness) that give meaning to their experience. (2) Second, the intervention focuses on looking back at important events from the past and ways of coping that have worked in the past in relation to the current cancer experience. Patients are encouraged to explore similarities and differences between past life challenges and the current cancer diagnosis, empowering them through reminding them of their emotional strengths and helping them discover a broader perspective and meaning of how they react to cancer and its treatment. (3) Third, the intervention includes talking about life objectives and values that give life meaning while also accepting the limits that come with sickness, such as the fact that life is short. Patients are invited to adjust their past goals and learn from their cancer experience. Ways of fulfilling these new goals are examined, and patients are encouraged to envision the possibility of their lives being shortened and explore related fears (e.g., pain, separation, loss of self, burial), envision an “acceptable death”, and identify unfinished business. If they are not willing to address the topic of death, discussions can focus on the possibility of a future cancer recurrence or progression and associated fears [30]. The MMi manual can be accessed by contacting the author of the intervention [11].

MMi sessions were conducted weekly for 3–4 weeks at peoples' home or in the hospital, depending on preference. They were led by one of four bilingual mental health professionals (a nurse, social worker, or psychotherapist) with 1–4 years of clinical oncology experience. Sessions were initiated within 1 week after randomization.

Each intervener received 42 h of theoretical and clinical training on the MMi by MH, including exercises and role‐playing. Initial patient sessions were audio‐recorded and reviewed until three consecutive complete interventions for each intervener corresponded to at least 80% of MMi tasks on the Audit Grid [11]. Supervision was provided weekly throughout the study and interventions were audited for treatment adherence by two Research Assistants (RAs) independent from recruitment.

#### 2.4.2 Attention Control Group

AC participants were assigned to visits with one of two empathic listeners, conducted at home or in hospital according to patient preference and on the same weekly schedule as the EG. These empathic listeners did not have a professional health care background and were not working in recruiting hospitals in parallel supportive roles. They received 32 h of training (with initial recording audits) to provide basic ingredients for fostering a therapeutic alliance (i.e., trust, warmth, empathy, neutrality, and authenticity) without initiating discussions about meaning or perspective‐taking (e.g., interpreting one's feelings and thoughts, illness experiences, or meaning in life), and instead focusing on what is currently happening. Visits were audio‐recorded, and their content was reviewed for training purposes until three consecutive AC interventions for each intervener were deemed acceptable (i.e., < 20% of tasks on the MMi Audit Grid for treatment adherence [11] for each participant, ensuring that AC visits did not overlap with MMi content). Supervision was provided weekly. Interventions were audited for treatment adherence by two RAs uninvolved in recruitment.

#### 2.4.3 Usual Care

UC referred to the usual oncological care people received at their affiliated hospital. A typical cancer care team includes medical, surgical, and radiation oncologists, plus pathologists, radiologists, and specialized nursing staff. Their combined skills support more coordinated, effective care. The group meets on a routine basis to discuss patients, share findings, and set plans aimed at better outcomes and experiences. Usual care also includes allied professionals (e.g., pharmacists, physiotherapists, occupational therapists, speech‐language pathologists, dietitians/nutritionists). Recruitment centers were large university‐affiliated teaching hospitals with well‐established psychosocial oncology services (i.e., psychiatrists, psychologists, social workers, nurses, and volunteers) that did not use a structured existential or meaning‐based approach.

### 2.5 Other Support

All participants were free to use hospital‐ or community‐based supports. Use of anxiolytic or antidepressant medication and psychosocial support (individual, couple, and/or group; frequency/duration of services) were tracked in all groups throughout the study by questionnaire and through a chart review and considered as potential confounders in analyses.

### 2.6 Treatment Adherence

MMi and AC sessions were audio‐recorded. After the initial fully audited training period, sessions of 20% randomly selected EG participants for each intervener and AC participants for each listener was each audited by two audit RAs (different than recruiting RAs). As part of audit quality control, MH audited a random 10% of audits to ensure proper conduct. Adherence consisted of MMi corresponding to ≥ 80% of overall tasks on the Audit Grid [11] for each participant, for each intervener and for each reviewer. The same MMi Audit Grid was used for AC visits, with adherence corresponding to < 20% of tasks for each participant, with therapeutic alliance tasks considered acceptable for the AC group. Further training was provided if kappa reliability was < 0.7 or a reviewer found that the MMi did not correspond to > 80% and the AC to < 20% of overall tasks on the Audit Grid for a participant. A consensus meeting was planned with both auditor RAs, MH and the Research Coordinator when this happened. These criteria and process were also used for the sessions audited as part of audit quality control. Audit RAs had a minimum of 1 year of clinical experience in a supportive role and were trained in the MMi approach. They were present during the theoretical MMi intervener training and reviewed audiotapes with MH until three of their consecutive sessions in the EG and AC respectively for each rater reached 0.70 inter‐rater reliability with MH's ratings. Randomization for RA audit and the audits of audit quality were carried out independently for EG and AC in blocks of 10 via the central randomization system, ensuring regular auditing of sessions throughout the trial.

### 2.7 Contamination

Several procedures were used to avoid contamination. MMi and AC intervenors were not clinical staff at study sites. MMi interveners did not discuss the intervention with recruiting centers' staff or AC staff and there was no presentation of the MMi to clinicians locally. The AC interveners had no prior knowledge of the MMi and all intervenors (MMi and AC) did not discuss clinical encounters within this project. Since MH is a clinical psychologist at one of the recruiting centers, she abstained from seeing people in the trial and referred them to other Psychosocial Oncology Program members if needed. Patients in the EG were asked upon randomization allocation not to discuss the intervention with other patients and staff, with questions in all follow‐up questionnaires to assess this (i.e., asking UC and AC if anyone spoke to them about the intervention content, asking EG patients if they discussed the intervention with other patients and/or staff, and if so, to provide names of people they spoke to, to assess whether they were in one of the control groups). We audited MMi and AC sessions using the audit grid, modified in the AC arm to rate the number of times empathic listeners used the concept of meaning‐making or perspective‐taking in their visits.

### 2.8 Outcome Measures

Measures were selected based on their psychometric properties, self‐administration, validation in French and English, and use in advanced cancer. Participants had a 1‐week delay to complete the questionnaires if needed for treatment side‐effect recovery and received up to three scripted reminder calls.

#### 2.8.1 Primary Outcome: Meaning in Life

Meaning in life was measured by the 8‐item FACIT‐Sp‐12 Meaning/Peace subscale [16, 17]. This 8‐item subscale focuses on having found meaning (which is what the MMi is intended to affect), has good psychometric properties (internal consistency: 0.81; test‐retest reliability: 0.80; convergent validity with QoL (FACT‐G) and divergent validity with a depression scale), is able to sensitively detect changes over time (with normally distributed scores and without ceiling or floor effects), and a minimally clinically important difference of 1.85–2.88 for the full FACIT‐Sp scale [31] It has been used in other similar studies of existential interventions in advanced cancer.

#### 2.8.2 Secondary Outcomes

Existential Wellbeing, overall QoL, and Physical Wellbeing (exploratory hypothesis) were measured by MQOL subscales. The MQOL [18, 19, 20] was developed to measure QoL in individuals living with a life‐threatening illness in 4 domains: physical, psychological, existential and support. Psychological distress (anxiety and depression) was measured by the Hospital Anxiety and Depression Scale (HADS) [21, 22] Total score, with a score of ≥ 16 customarily used as a threshold for clinically significant distress [32]. Posttraumatic growth was measured by the Posttraumatic Growth Inventory (PTGI). The PTGI was developed to measure growth and other favorable outcomes following trauma [23].

### 2.9 Sociodemographic and Medical Information

We created a questionnaire to collect sociodemographics, religious beliefs, perceived life threat (11‐point numerical rating scale from 0‐Not at all to 10‐Completely), experience with cancer among family/friends, current physical discomfort, and functioning (ECOG performance status). At each evaluation point, participants indicated whether they used professional psychological support (individual, couple or group psychotherapy, psychiatric medication) outside the protocol as well as additional emotional support from health care worker(s). Illness/treatment information was gathered through medical charts at each evaluation point advanced disease diagnosis date, disease stage III/IV, disease progression/remission, treatment type (surgery/chemotherapy/radiotherapy).

### 2.10 Sample Size

A full RCT required 157 patients in each arm for a total of 471 patients to achieve 80% statistical power, using two independent two‐tailed two‐sample *t*‐tests at 0.025 significance level (Bonferroni correction). The sample size estimate was based on a small to medium effect size of 0.35 on the primary outcome, meaning in life on the FACIT‐Sp‐12 meaning/peace subscale, between the experimental group and each of the two controls (experimental group vs. attention control and experimental group vs. usual care control), likely to be clinically significant and within the range of effect sizes reported for other psychosocial interventions [33] including cancer patients. The sample size was calculated using PASS version 13 [34].

### 2.11 Randomization, Allocation, and Blinding

Participants were randomized by trial arm (1:1:1 ratio) and, once assigned, by intervener (1:1 ratio) for the EG and AC groups. All researchers, research staff, the interveners who delivered the MMi and AC listeners were blind to the randomization sequence, carried out in random permuted blocks of 2, 4, and 6 using a web‐based central randomization system (CRS) from an office unconnected with study conduct. Randomization was stratified by: (1) recruiting hospital and (2) sex (male/female). Group assignment took place after baseline questionnaires were received and checked for completeness.

MMi interveners, AC listeners, health care providers, investigators, and RAs auditing for treatment adherence were blind to questionnaire data during the trial (only recruiting RAs saw the data, for data checking and data entry). While participants were not blinded to their assigned group, details about the intervention were kept minimal, omitting words “meaning in life” or “Meaning‐Making intervention” from recruitment material and consent forms to avoid expectation bias (24).

### 2.12 Statistical Methods

Data were analyzed using SAS version 9.4. Baseline demographic and clinical characteristics were summarized to describe the three treatment groups, using mean or medians for continuous data and proportions for categorical data.

To test our primary and secondary hypotheses, we compared treatment between‐group unadjusted scores at 2 months post‐randomization, using independent two‐sample *t*‐tests on primary and secondary outcomes. We then compared treatment between‐group primary and secondary outcomes at 2, 4, and 6 months post‐randomization, using a mixed effect linear regression model to adjust for baseline characteristics (i.e., baseline level for each analyzed outcome, time between advanced disease diagnosis and baseline questionnaire completion, any psychotherapy (yes/no) and psychiatric medication (yes/no) outside the protocol, and ECOG status (scores 1–4)) and random time effect.

With the intention of fully using the data, we conducted analyses to explore who benefits most from the intervention e.g., cancer stage, gender, degree of perceived life‐threat, high initial psychological distress (HADS ≥ 16), initial meaning in life (FACIT‐Sp‐12), and degree of perceived physical wellbeing (MQOL Physical Wellbeing item), using linear regression for measures at 2 months post‐randomization and mixed effect linear models to include outcome measures at 2, 4, and 6 months post‐randomization all together.

Data was analyzed on an intention‐to‐treat basis. People who dropped out of the treatment were asked to complete all outcome measures (or at least the primary outcome, if possible) and their dropout reasons were tracked. Missing data was imputed using multiple imputation with the Markov Chain Monte Carlo method [35]. There were no interim analyses.

## 3 Results

Baseline demographic and clinical characteristics are found in Table 1 and the Consort Flow Diagram is found in Figure 1. Participants were mostly female (65.7%), married (63.7%), Caucasian (80.2%), had a university education (48.5%), and had a mean age of 63.2 years (SD = 11.4). A total of 84.5% were newly diagnosed with cancer, 10.9% experienced advanced stage recurrence, and 4.6% had advanced stage progression. Stage IV was observed in 46.9%, Stage III in 38.1%, and advanced but unknown stage in 15.1%. The most common cancers were lung (49.2%), gynecological (20.3%), colon (8.5%), head and neck (6.8%), and breast (5.9%).

**TABLE 1:** Socio‐demographic and medical variables (*N* = 239).

		EG	AC	UC	*p*
	*N* (%)/Mean (SD)	*N* (%)/Mean (SD)	*N* (%)/Mean (SD)	*N* (%)/Mean (SD)	
Hospital					
Jewish general hospital	149 (62.3)	50 (20.0)	52 (21.8)	47 (19.7)	0.773
McGill university health center	90 (37.7)	30 (12.6)	28 (11.7)	32 (13.4)	
Sex					
Male	82 (34.3)	26 (10.9)	26 (10.9)	30 (12.6)	0.704
Female	157 (65.7)	54 (22.6)	54 (22.6)	49 (20.5)	
Age	63.2 (11.4)	63.4 (10.3)	63.6 (10.6)	62.7 (13.0)	0.001
Language					
English	137 (57.3)	42 (17.6)	44 (18.4)	51 (21.3)	0.269
French	102 (42.7)	38 (15.9)	36 (15.1)	28 (11.7)	
Marital status					
Married/Common‐law	151 (63.7)	51 (21.5)	49 (20.7)	51 (21.5)	
Divorced/Separated	42 (17.7)	17 (7.2)	14 (5.9)	11 (4.6)	0.502
Single (never married)	29 (12.2)	5 (2.1)	11 (4.6)	13 (5.5)	
Widowed	15 (6.3)	5 (2.1)	6 (2.5)	4 (1.7)	
Ethnicity					
Caucasian/Canadian	182 (80.2)	62 (27.3)	61 (26.9)	59 (26.0)	0.565
Other	45 (19.8)	15 (6.6)	12 (5.3)	18 (7.9)	
Education					
Elementary	7 (2.9)	2 (0.8)	4 (1.7)	1 (0.4)	
High school	72 (30.1)	19 (7.9)	23 (9.6)	30 (12.6)	0.43
CEGEP/vocational	41 (17.1)	17 (7.1)	15 (6.3)	9 (3.8)	
University undergraduate	45 (18.8)	14 (5.9)	16 (6.7)	15 (6.3)	
University graduate	71 (29.7)	27 (11.3)	21 (8.8)	23 (9.6)	
Individual income					
< 19,000/yr.	55 (25.9)	20 (9.4)	16 (7.5)	19 (9.0)	
20,000–39,000/yr.	52 (24.5)	16 (7.5)	16 (7.5)	20 (9.4)	
40,000–59,000/yr.	41 (19.3)	13 (6.1)	22 (10.4)	6 (2.8)	0.022
60,000–79,000/yr.	27 (12.7)	9 (4.2)	3 (1.4)	15 (7.1)	
80,000–99,000/yr.	16 (7.5)	5 (2.4)	8 (3.8)	3 (1.4)	
Over 100,000/yr.	21 (9.9)	7 (3.3)	8 (3.8)	6 (2.8)	
Work status now					
Employed full‐time	43 (18.3)	12 (5.1)	16 (6.8)	15 (6.4)	
Employed part‐time	15 (6.4)	10 (4.3)	3 (1.3)	2 (0.9)	
Unemployed	50 (21.3)	16 (6.8)	14 (6.0)	20 (8.5)	0.230
Studying full‐time	1 (0.4)	0 (0)	0 (0)	1 (0.4)	
Homemaker	18 (7.7)	8 (3.4)	5 (2.1)	5 (2.1)	
Retired	85 (36.2)	26 (11.1)	31 (13.2)	28 (11.9)	
Medical leave	23 (9.8)	5 (2.1)	11 (4.7)	7 (3.0)	
Current status					
Initial diagnosis	202 (84.5)	70 (29.3)	65 (27.2)	67 (28.0)	0.852
Progression to advanced stage	11 (4.6)	3 (1.3)	4 (1.7)	4 (1.7)	
Recurrence in advanced stage	26 (10.9)	7 (2.9)	11 (4.6)	8 (3.3)	
Stage upon study enrollment					
Stage III	91 (38.1)	32 (13.4)	30 (12.6)	29 (12.1)	0.956
Stage IV	112 (46.9)	38 (15.9)	37 (15.5)	37 (15.5)	
Not stage but advanced	36 (15.1)	10 (4.2)	13 (5.4)	13 (5.4)	
Cancer site					
Lung	116 (49.2)	37 (15.7)	35 (14.8)	44 (18.6)	
Colon	20 (8.5)	6 (2.5)	9 (3.8)	5 (2.1)	
Gynecological	48 (20.3)	21 (8.9)	17 (7.2)	10 (4.2)	
Breast	14 (5.9)	9 (3.8)	1 (0.4)	4 (1.7)	
Head & neck	16 (6.8)	5 (2.1)	5 (2.1)	6 (2.5)	0.309
Hematology	4 (1.7)	1 (0.4)	0 (0)	3 (1.3)	
GI	7 (3.0)	1 (0.4)	3 (1.3)	3 (1.3)	
Urologic	7 (3.0)	2 (0.8)	3 (1.3)	2 (0.8)	
Rectal	1 (0.4)	1 (0.4)	0 (0)	0 (0)	
Musculoskeletal	2 (0.8)	1 (0.4)	0 (0)	1 (0.4)	
Pancreatic	1 (0.4)	0 (0)	0 (0)	1 (0.4)	
ECOG (self‐rated)					
0	73 (30.7)	27 (11.3)	21 (8.8)	25 (10.5)	
1	92 (38.7)	31 (13.0)	33 (13.9)	28 (11.8)	0.561
2	54 (22.7)	17 (7.1)	18 (7.6)	19 (8.0)	
3	16 (6.7)	2 (0.8)	8 (3.4)	6 (2.5)	
4	3 (1.3)	2 (0.8)	0 (0)	1 (0.4)	

FIGURE 1CIHR MMi full trial in advanced cancer. Consort flow diagram including current full trial (Feb‐2016‐Apr‐2020) and pilot (Oct‐2012‐Jun‐2013).

**Figure:**
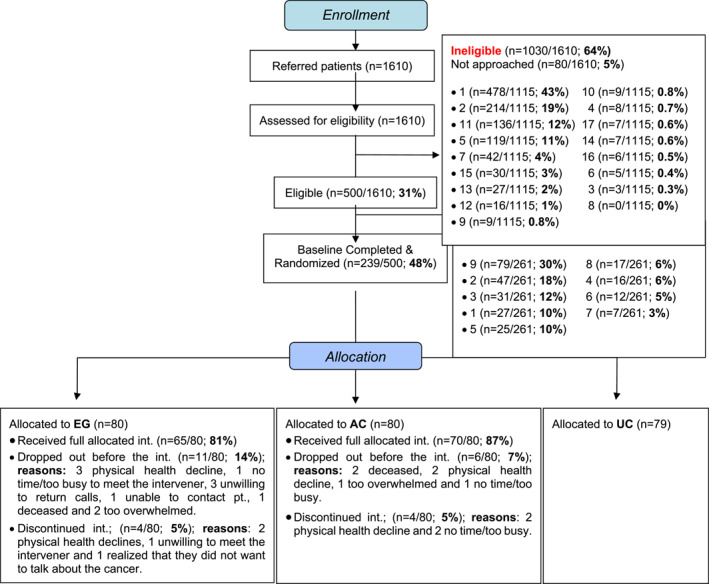

**Figure:**
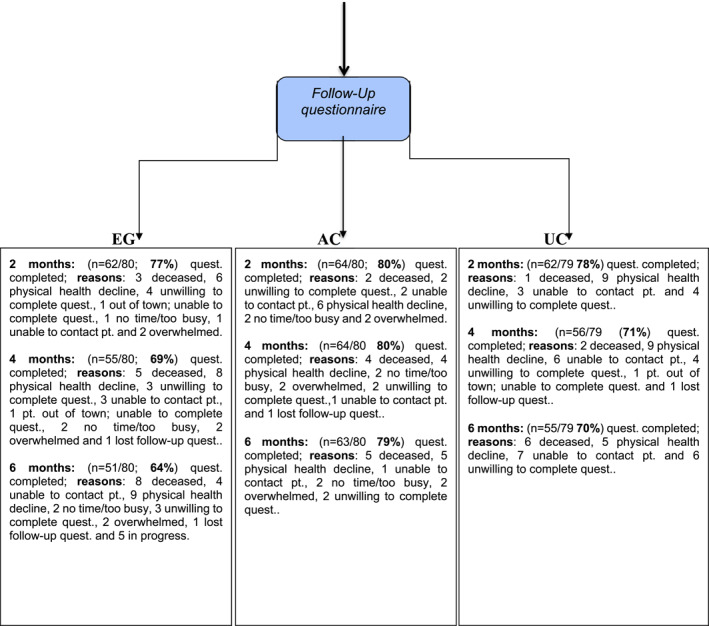

**Figure:**
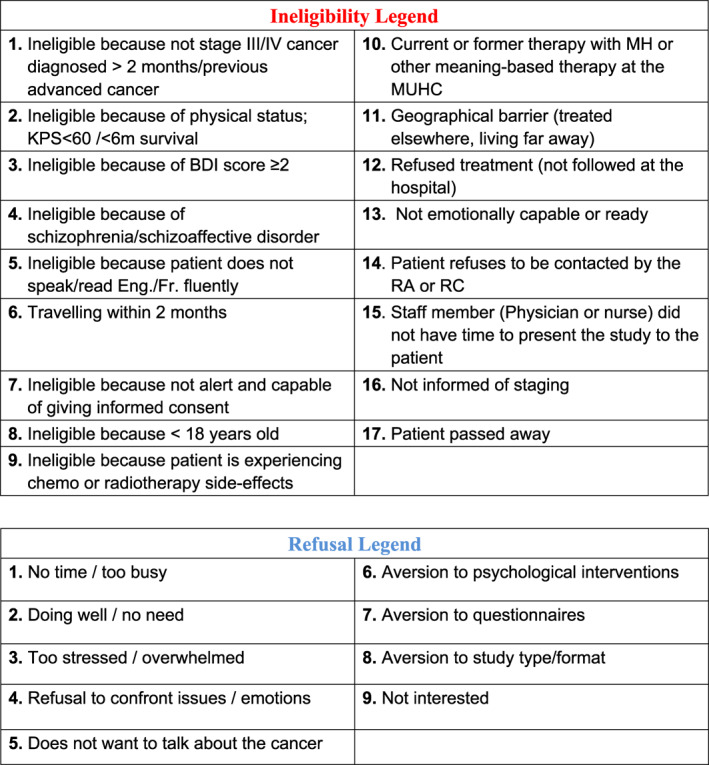

### 3.1 Intervention Adherence and Contamination

Eighty‐eight percent (15/17) of audited MMi sessions met the audit criteria while 100% of AC sessions did. Quality control audits were conducted on 21% of EG and 15% of AC interventions. Overall inter‐rater reliability on quality control audits was good (kappa = 0.89, *p* < 0.001, CI 0.83–0.95). In terms of contamination, no patient reported speaking to other patients and/or staff (for the EG) or hearing from other patients and/or staff about the intervention (for the AC and UC) (See Table 2).

**TABLE 2:** Retention rates and intervention parameter comparison between groups (*n* = 239).

Follow‐up (months)	Group allocation	*N* (%)	Overall retention (%)
2 months	EG	62/80 (77.5)	188/239 (79)
	AC	64/80 (80.0)	
	UC	62/79 (78.5)	
4 months	EG	55/80 (68.8)	175/239 (73)
	AC	64/80 (80.0)	
	UC	56/79 (70.9)	
6 months	EG	51/80 (63.8)	169/239 (71)
	AC	63/80 (78.8)	
	UC	55/79 (69.6)	

**Intervention**	**Group allocation**	***N* (%)**	
Received full allocated intervention	EG	65/80 (81.3)	—
	AC	70/80 (87.5)	—
Completed intervention within 3–4 weeks	EG	51/65 (78.5)	—
	AC	59/70 (84.2)	—
Completed intervention before the 2‐months follow‐up questionnaires	EG	55/65 (84.6)	—
	AC	57/70 (81.4)	—

**Intervention**	**Group allocation**	**Min (sd)/session (sd)/days (sd)**	**Difference (%)**
Avg intervention length (min) (SD)	EG	237.31 (87.0)	19.7
	AC	190.53 (62.6)	
Avg session length (min) (SD)	EG	80.79 (25.5)	24.8
	AC	60.78 (20.3)	
Avg. sessions per patient (SD)	EG	3.15 (0.4)	4.4
	AC	3.01 (0.3)	
Avg. Intervention durations (days) (SD)	EG	26.98 (14.1)	4.1
	AC	28.13 (20.5)	

Abbreviations: AC, Attention‐Control Group; EG, Experimental Group; min, minutes; sd, standard deviation.

### 3.2 Primary Efficacy Analyses

There were no statistically significant (*p* < 0.05) inter‐group differences in FACIT‐Sp‐12 Meaning subscale scores 2 months post‐randomization in independent two‐sample *t*‐tests (EG vs. UC *p* = 0.65; EG vs. AC *p* = 0.94) (means over time in Table 3; differences in Table 4), nor at 2, 4, and 6 months post‐randomization using a mixed effect linear regression model and adjusting for baseline characteristics and random effect of time (*p* = 0.55–0.99).

**TABLE 3:** Outcome scores over time (*N* = 239).

Outcome	Baseline mean (SD)	2 months mean (SD)	4 months mean (SD)	6 months mean (SD)
FACIT‐Sp‐12 meaning/peace^a^				
UC	20.2 (4.1)	19.7 (4.7)	19.5 (4.0)	19.9 (3.7)
AC	19.2 (3.9)	19.3 (4.1)	20.0 (3.9)	19.7 (3.7)
EG	19.7 (4.2)	19.2 (4.2)	18.7 (4.6)	18.8 (4.7)
MQOL existential^b^				
UC	6.2 (2.3)	6.5 (2.5)	6.4 (2.5)	6.9 (2.4)
AC	6.6 (2.4)	6.9 (2.0)	7.1 (1.8)	7.0 (2.2)
EG	6.5 (2.3)	6.4 (2.1)	6.3 (2.4)	6.6 (2.3)
HADS total^c^				
UC	12.3 (6.6)	11.8 (7.5)	12.9 (8.3)	12.8 (8.2)
AC	12.4 (7.6)	12.2 (7.1)	10.5 (5.8)	11.4 (7.1)
EG	13.0 (7.0)	13.8 (8.1)	13.3 (8.1)	13.7 (7.8)
MQOL total^d^				
UC	6.9 (1.8)	6.8 (1.9)	6.7 (1.7)	6.9 (1.7)
AC	6.9 (1.6)	6.8 (1.4)	7.0 (1.4)	6.9 (1.5)
EG	6.9 (1.5)	6.6 (1.7)	6.5 (1.7)	6.5 (1.9)
PTGI^e^				
UC	55.5 (28.7)	53.4 (25.5)	52.8 (24.0)	57.9 (23.3)
AC	53.7 (23.5)	55.8 (24.0)	58.8 (22.8)	58.3 (26.1)
EG	50.0 (24.9)	52.6 (24.7)	52.8 (21.6)	51.9 (23.9)

^a^ higher score = greater meaning in life.

^b^ higher score = greater existential wellbeing.

^c^ higher score = greater symptoms of anxiety and depression.

^d^ higher score = better quality of life.

^e^ higher score = more post‐traumatic growth.

Abbreviations: AC, Attention‐Control Group; EG, Experimental Group; FACIT‐Sp‐12, Functional Assessment of Chronic Illness Therapy—Spiritual Wellbeing 12; HADS, Hospital Anxiety and Depression Scale; MQOL, McGill Quality of Life; PTGI, Post‐Traumatic Growth Inventory; UC, Usual Care.

**TABLE 4:** Between group differences and 95% confidence intervals (CI) at 2 months (primary endpoint).

Outcome measure	EG versus UC				EG versus AC			
	Mean	SE	CI lower bound	CI upper bound	Mean	SE	CI lower bound	CI upper bound
FACIT‐Sp 12 meaning and peace subscale	−0.34	0.73	−1.77	1.09	0.05	0.72	−1.36	1.46
MQOL existential wellbeing subscale	−0.24	0.32	−0.86	0.38	−0.24	0.33	−0.88	0.40
HADS total	1.03	1.24	−1.40	3.46	0.81	1.27	−1.67	3.29
MQOL total	−0.19	0.28	−0.73	0.35	−0.22	0.28	−0.76	0.32
PTGI	−1.06	4.26	−9.40	7.28	−3.61	4.37	−12.17	4.95

Abbreviations: AC, Attention‐Control Group; EG, Experimental Group; FACIT‐Sp‐12, Functional Assessment of of Chronic Illness Therapy—Spiritual Wellbeing 12; HADS, Hospital Anxiety and Depression Scale; MQOL, McGill Quality of Life; PTGI, Post‐Traumatic Growth Inventory; UC, Usual Care.

### 3.3 Secondary Analyses

There were no statistically significant inter‐group differences for any of the secondary outcome measures (i.e., MQOL Existential Wellbeing subscale; HADS; MQOL Total; PTGI) at 2 months post‐randomization in independent two‐sample *t*‐tests (*p* = 0.41–0.80) (Tables 3 and 4), nor at 2, 4, and 6 months post‐randomization using a mixed effect linear regression model adjusting for baseline characteristics and random effect of time (*p* = 0.30–0.99).

### 3.4 Exploratory Analyses

In exploratory analyses focused on outcomes of meaning in life (FACIT‐Sp‐12) and post‐traumatic growth (PTGI), stage III EG participants seemed to present higher meaning in life on the FACIT‐Sp‐12 and post‐traumatic growth on the PTGI 2 months post‐randomization than patients in the AC (est. 4.72, SE 2.23, *p* = 0.04 and est. 30.39, SE 13.43, *p* = 0.02). Applying a Bonferroni correction for number of comparisons (*n* = 30) with a *p* value of 0.002 renders these differences non‐significant. No other significant differences were found on exploratory analyses.

## 4 Discussion

This study is the first to evaluate the efficacy of the Meaning‐Making Intervention (MMi) in a large sample of patients newly diagnosed with advanced cancer. The trial found no significant effects of the intervention on planned primary and secondary outcomes compared to an attention control or usual care at 2 months, with similar patterns at later assessments. Exploratory analyses suggest higher post‐traumatic growth in patients with locally advanced stage III cancer at 2 months post‐randomization compared to people in the AC group (approximately 1 month post‐intervention), but these effects cannot be considered significant when applying a Bonferroni correction and should be interpreted cautiously.

Features of our study design increase confidence that the observed null effects reflect the intervention as delivered (3‐4 sessions over ∼4 weeks) rather than methodological artifact. These features include: a three‐arm RCT with both AC and UC comparators; masking of condition labels and description to minimize expectancy effects; continuous and randomized fidelity auditing; low contamination; and blinded data collection.

Prior work on meaning‐centered and related existential interventions has generally reported small effects on meaning and mood, often with longer protocols or samples already presenting with high levels of distress [1, 2, 36, 37, 38, 39]. Differences in *dose* (our brief format vs. 6–7 sessions over 7–12 weeks, or multi‐month protocols) and *participant selection* (unselected vs. distress‐selected samples) may account for the discrepancy, although some trials also proposed the intervention to all. One meta‐analysis also suggests greater session numbers are associated with stronger effects on depression and anxiety [36]. Notably, across broader psychosocial intervention meta‐analyses, cancer stage has not consistently moderated outcomes [1, 36]; our stage III signal therefore remains hypothesis‐generating rather than definitive.

### 4.1 Implications for Future Research and Practice

Future research might test the effect of the MMi specifically in people who are experiencing elevated existential distress or extend the number or spacing of sessions. It would also be useful to examine how meaning‐making processes intersect with psychological distress, physical symptom burden, and socioeconomic determinants of health, and to evaluate potential stage‐specific effects. On the basis of this single trial, routine integration of the MMi into practice is not supported; however, the need for scalable approaches to existential distress remains clear [1, 2].

### 4.2 Study Limitations

Amid this study's rigorous design, it presents several limitations. First, its sample size was smaller than anticipated. However, the null results obtained are unlikely to reflect power alone, considering that each arm had 59‐60 participants and group means showed little difference. Second, as is typical in intervention research, most participants were women, emphasizing the importance of outreach strategies to recruit men. Third, lung cancer was overrepresented, and different heterogeneity in tumor types and treatment courses may influence perceived threat and meaning. Fourth, although the intervention's adaptability was a strength and supports personalization, it might have introduced variability in how existential themes were addressed. Fifth, exploratory analyses are vulnerable to type I error and should be read cautiously. Finally, while randomized controlled trials are well suited to answer the question of efficacy, they are less informative about why an intervention succeeds or fails, limiting guidance on how best to refine the MMi or its delivery context.

## 5 Conclusion

In this three‐arm randomized controlled trial, the MMi did not produce improvements on the primary or secondary outcomes compared with AC or UC. There may be some indications of a signal for benefit for patients with stage III cancer, which warrant follow‐up but cannot be considered definitive. Future work should prioritize targeting, dose, timing, and contextual moderators to clarify when, and for whom, meaning‐focused approaches are most effective.

## Author Contributions

Melissa Henry and Robin Cohen served as the principal investigators, leading the conceptualization and design of the study. They jointly reviewed the analyses and co‐drafted the manuscript. Melissa Henry was responsible for overall project administration, supervision, and training. Daren Heyland and Robert Platt contributed to RCT design and interpretation. Xun Zhang planned and carried out the statistical analyses. Margarida Costa, Cassy Shitong Wang, and Clara Bolster‐Foucault were responsible for the day‐to‐day project administration and oversaw data entry. Walter Gotlieb, Susie Lau, Carol‐Ann Vasilevsky, Michael Hier, Nader Sadeghi, Khalil Sultanem, Gerald Batist, and Jason Agulnik enabled recruitment. Melissa Henry, S Robin Cohen, Daren Heyland, Robert Platt and Xun Zhang were involved in the study design. All reviewed and critiqued manuscript drafts and approved the final version.

## Funding

This study was supported by Canadian Institutes of Health Research (Grants 340947 and 258431).

## Ethics Statement

Ethics approval was obtained from the Research Ethics Committee of the Integrated Health and Social Services University Network for West‐Central Montreal #MM‐JGH‐15–100, the McGill University Health Center Centre for Applied Ethics #MPE‐CUSM‐15–242, and the McGill University Faculty of Medicine and Health Sciences Institutional Review Board #A00‐M71‐12B.

## Conflicts of Interest

The authors declare no conflicts of interest.
